# Integrative analysis with ChIP-seq advances the limits of transcript quantification from RNA-seq

**DOI:** 10.1101/gr.199174.115

**Published:** 2016-08

**Authors:** Peng Liu, Rajendran Sanalkumar, Emery H. Bresnick, Sündüz Keleş, Colin N. Dewey

**Affiliations:** 1 Department of Biostatistics and Medical Informatics, University of Wisconsin, Madison, Wisconsin 53706, USA;; 2 Department of Cell and Regenerative Biology, UW-Madison Blood Research Program, Carbone Cancer Center, University of Wisconsin School of Medicine and Public Health, University of Wisconsin, Madison, Wisconsin 53705, USA;; 3 Department of Statistics, University of Wisconsin, Madison, Wisconsin 53706, USA;; 4 Department of Computer Sciences, University of Wisconsin, Madison, Wisconsin 53706, USA

## Abstract

RNA-seq is currently the technology of choice for global measurement of transcript abundances in cells. Despite its successes, isoform-level quantification remains difficult because short RNA-seq reads are often compatible with multiple alternatively spliced isoforms. Existing methods rely heavily on uniquely mapping reads, which are not available for numerous isoforms that lack regions of unique sequence. To improve quantification accuracy in such difficult cases, we developed a novel computational method, prior-enhanced RSEM (pRSEM), which uses a complementary data type in addition to RNA-seq data. We found that ChIP-seq data of RNA polymerase II and histone modifications were particularly informative in this approach. In qRT-PCR validations, pRSEM was shown to be superior than competing methods in estimating relative isoform abundances within or across conditions. Data-driven simulations suggested that pRSEM has a greatly decreased false-positive rate at the expense of a small increase in false-negative rate. In aggregate, our study demonstrates that pRSEM transforms existing capacity to precisely estimate transcript abundances, especially at the isoform level.

Transcriptome profiling by high-throughput next-generation DNA sequencing (RNA-seq) comprehensively measures transcript abundances within a sample of cells at a given moment (Mortazavi et al. 2008; Wang et al. 2009). It has been applied to diverse problems, such as identifying differentially expressed genes or isoforms, cataloging long intergenic noncoding RNAs, and detecting gene fusions in diseased tissues (Maher et al. 2009; Cabili et al. 2011; Trapnell et al. 2013). A typical RNA-seq experiment first involves the selection of a fraction of transcripts of interest, followed by fragmentation, reverse transcription, and high-throughput sequencing. Millions of reads from sequencing are mapped back to a reference genome or transcriptome and quantified to estimate the expression of isoforms and genes (Mortazavi et al. 2008). Due to alternative splicing and the repetitiveness of genomic sequence (Treangen and Salzberg 2012), RNA-seq reads that map to multiple isoforms of the same gene or to multiple genes at different genomic locations are prevalent. For example, in an RNA-seq data set from the human erythroleukemia cell line K562 (Djebali et al. 2012), 94% of expressed protein-coding genes have reads mapping to multiple isoforms, and more than 20% of expressed protein-coding genes share reads between each other (Fig. 1A). These multimapping reads cause ambiguities in the mapping step and therefore complicate abundance estimation at both the isoform and gene levels.

**Figure 1.**
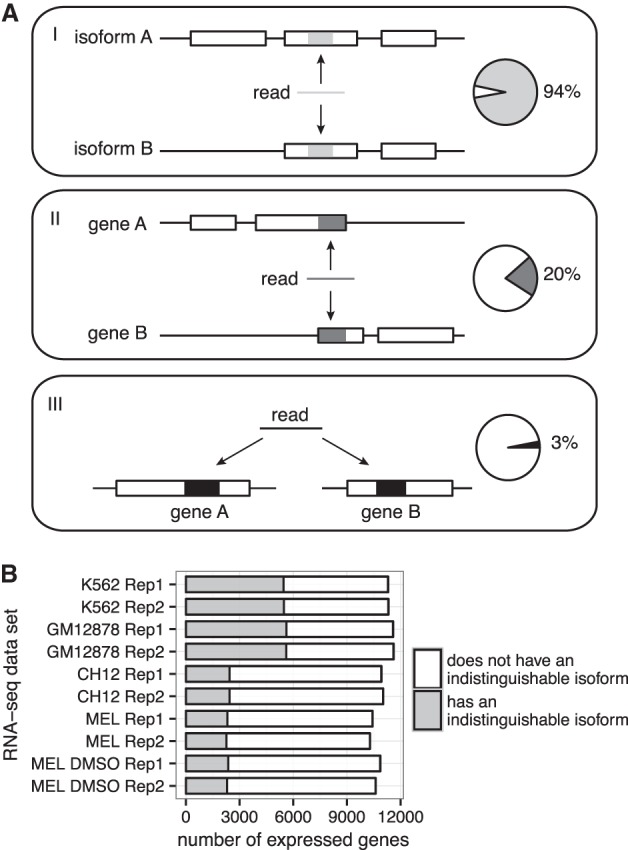
Multimapping reads are prevalent in human and mouse RNA-seq data. (*A*) Three classes of multimapping reads and the percentages of expressed genes (TPM ≥ 1) to which each class maps in the K562 replicate one data set. (*B*) Number of expressed genes that have indistinguishable isoforms. An indistinguishable isoform is one for which all potential RNA-seq fragments derived from it can align to other isoforms (for the calculations of distinguishability, see section I.B in the Supplemental Material). An expressed gene is required to have abundance of one TPM or more. Data are from the two RNA-seq replicates (“Rep1” and “Rep2”) of each of the human cell lines K562 and GM12878 and the mouse cell lines CH12, MEL, and MEL DMSO.

Several strategies exist for handling multimapping reads. Cufflinks divides multimapping reads equally among candidates by default (Trapnell et al. 2010). ERANGE employs a ‘rescue’ approach to allocate multimapping reads to genes in proportion to the number of reads uniquely mapping to them (Mortazavi et al. 2008). RSEM and eXpress adopt the expectation-maximization (EM) algorithm, in which alternating steps are taken between assigning reads fractionally to isoforms based on current model parameters (the expectation step) and updating model parameters (which include isoform abundances) according to the read assignments (the maximization step) (Li et al. 2010; Roberts and Pachter 2013).

Despite the success of all these approaches, allocation of multimapping reads relies heavily on the information provided by uniquely mapped reads. When this information is unavailable such as for “indistinguishable” isoforms, which lack unique sequence (Fig. 1A, isoform B in panel *I*), it is often difficult to accurately estimate abundances with current methods. We found that there are 20,738 indistinguishable isoforms from human protein-coding genes and 7040 from mouse, accounting for 14% and 9% of all the isoforms, respectively (Supplemental Figs. S1B, S2B). Further, >45% of expressed protein-coding genes in human and >20% in mouse have an “indistinguishable” isoform (Fig. 1B; Supplemental Figs. S1D, S2D). If one considers that RNA-seq read distributions across isoforms are often nonuniform (Wang et al. 2009), the number of expressed isoforms lacking uniquely mapped reads can be even higher because a unique exon or junction from an expressed isoform may not necessarily be sequenced. Such substantial numbers of indistinguishable isoforms present a major challenge for the task of allocating multimapping reads.

In this work we developed and characterized a novel strategy for further ameliorating the multimapping issue that uses information from external complementary data. Such an integrative approach has been recently considered for multimapping issues concerning ChIP-seq data (Zeng et al. 2015). Studies of the relationships between expression and genomic signals measured by a variety of high-throughput assays have indicated that such signals are informative regarding transcript abundances. For example, the relationship between RNA Polymerase II (Pol II) ChIP-seq data and RNA-seq data has been investigated in the fly (Gan et al. 2010) and mouse (Deng et al. 2011). In both species, a positive correlation was observed between Pol II occupancy around transcription start sites (TSSs) and expression levels of the corresponding genes. Similarly, histone modifications have been suggested to mark transcription initiation sites (Guenther et al. 2007). In mouse Th2 cells, gene expression levels were found to be correlated with H3K9/14ac ChIP-seq signals (Hebenstreit et al. 2011). Furthermore, ChIP-seq data of histone modifications have been used to predict expression levels of genes (Karlić et al. 2010; Dong et al. 2012).

Our strategy has been implemented in a computational framework named ‘prior-enhanced RSEM’ (pRSEM). This framework integrates RNA-seq and other data types relevant to the sample of interest for the task of RNA-seq transcript quantification. This integration is accomplished through the Bayesian statistical technique of placing a prior probability distribution (herein after simply referred to as the “prior”) over transcript abundances, which we establish from the other data types. In principle, the prior can be derived from a variety of sources so long as they are informative with regard to transcript abundances. Here, we describe pRSEM's workflow and demonstrate that ChIP-seq data of Pol II and histone modifications serve as effective sources of prior knowledge within the pRSEM framework. We examine whether pRSEM's allocation of multimapping reads agrees with Pol II occupancy data and validate pRSEM's accuracy with both RAMPAGE data and qRT-PCR experiments. Through data-driven simulations, we compare the performance of pRSEM with that of eXpress and RSEM. Both the experimental and simulation results provide evidence that pRSEM produces superior quantifications, particularly for low abundance and unexpressed isoforms.

## Results

### A novel RNA-seq quantification method using a Pol II ChIP-seq data-derived prior

The pRSEM framework was built upon the RSEM statistical model for RNA-seq quantification (Li et al. 2010; Li and Dewey 2011) with two novel features. First, pRSEM places a prior distribution over transcript abundances, the parameters for which are estimated from the RNA-seq data itself. Second, transcripts are partitioned using an additional data source, with a different prior distribution induced over each partition. Intuitively, we wish to make partitions such that transcripts with similar abundances fall within the same partition and therefore that the prior distribution may be as informative as possible. We explored a variety of partition models but did not find any that were overwhelmingly superior (Supplemental Fig. S3). Hence, for the sake of simplicity and interpretability, we selected a two-partition scheme that aims to separate expressed from unexpressed transcripts. As we will show later in this section, Pol II ChIP-seq data accurately predicts transcript expression status, and therefore, we initially built pRSEM around this data type. Note that the pRSEM framework is general and allows for an arbitrary number of partitions, which can be derived from any type of external data.

**Figure 2.**
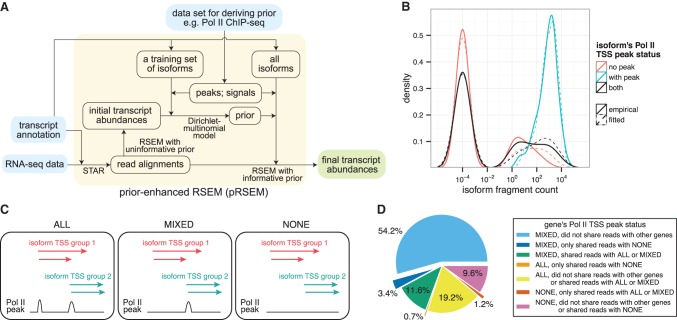
Pol II TSS peak data are informative for deriving a pRSEM prior. (*A*) pRSEM's workflow consists of inputs (light blue), intermediate steps (light orange), and output (green). (*B*) Empirical (solid lines) and fitted (dashed lines) distributions of fragment counts for isoforms in a pRSEM training set, stratified by Pol II TSS peak status. A small fractional count (10^−4^) was added to each isoform's fragment count so that isoforms with zero fragments could be depicted on a log scale. (*C*) Three types of gene Pol II TSS peak status. (*D*) Percentages of expressed genes classified by Pol II TSS peak status and multiread sharing. All fragment counts and percentages were calculated by RSEM on the K562 RNA-seq replicate one data set.

The workflow of pRSEM consists of three steps: (1) processing external data sets, (2) learning prior parameters, and (3) applying prior parameters for abundance estimation (Fig. 2A). To process external Pol II ChIP-seq data, for instance, pRSEM has utilized the ENCODE standard protocol (SPP peak caller and IDR pipeline) (Landt et al. 2012) to obtain Pol II peaks (and signals, depending on the partition model). Precomputed ChIP-seq peaks can also be provided to pRSEM to speed up the process. In the second step, a training set of isoforms, to which ChIP-seq reads and peaks can be uniquely assigned, is built and partitioned (for the construction of a training set, see section II.B in the Supplemental Material). The default two-partition model in pRSEM is to place isoforms with a Pol II peak in one partition and those without such a peak in the other. An isoform is defined to have a Pol II peak if a ChIP-seq peak overlaps with the 500-nucleotide (nt) flanking region of its TSS (for simplicity, we refer to such a peak as a “Pol II TSS peak”). In addition, pRSEM provides four other types of partition models that categorize isoforms by both Pol II peaks and signals (see section II.B in the Supplemental Material). Unless mentioned otherwise, we will use the default two-partition scheme to illustrate pRSEM in this work. After building the training set, an ENCODE standard pipeline of STAR (Dobin et al. 2012) and RSEM (Li and Dewey 2011) is employed to obtain estimates of read counts for each isoform. Through a Dirichlet-multinomial model, pRSEM learns a Dirichlet prior parameter for each partition. Lastly, all isoforms are partitioned in the same way as those in the training set, and prior parameters are assigned to isoforms in the corresponding partition. RSEM's Gibbs sampling routine is then run with the learned prior parameters, which serve as “pseudo” read counts for the isoforms. Compared with RSEM, pRSEM (Supplemental Tables S1, S2) uses the same amount of memory and takes ∼48% more time when quantifying human RNA-seq data (Supplemental Table S3).

A good data set for deriving a pRSEM prior must fulfill two requirements: (1) It can be used to partition the training set into groups that have distinct RNA-seq read count distributions, and (2) the data set is informative for a large fraction of target genes. We find that Pol II ChIP-seq data meets both requirements. To examine the first requirement, we built a training set for human cell line K562 based on ENCODE RNA-seq and ChIP-seq data sets (Supplemental Tables S1, S2). The training set contained 172 isoforms with a Pol II TSS peak and 897 isoforms without a peak. The RNA-seq fragment counts of these two groups of isoforms had strikingly distinct distributions (Fig. 2B). Fragment counts of the 172 isoforms with a Pol II TSS peak ranged from one to tens of thousands with a mode around 1000. In contrast, the majority of the 897 isoforms without a Pol II TSS peak did not have a single fragment, and the small fraction with fragments had counts ranging from one to 100. All three modes from the two partitions were observed in the joint distribution of fragment counts (Fig. 2B), demonstrating that Pol II TSS peak status properly dissects the joint distribution and is a good indicator of isoform abundance.

The prior parameters of pRSEM's Dirichlet-multinomial model learned from the K562 data were 0.60 and 0.04, corresponding to the pseudofragment counts of isoforms in the with-peak and without-peak partitions, respectively. This learned prior distribution fits the data well (Fig. 2B). We found similarly good fits with data sets from other human and mouse cell lines (Supplemental Fig. S4), indicating that Pol II TSS peak status is an effective feature with which to partition isoforms and that pRSEM's Dirichlet-multinomial model correctly captures the fragment count distributions of the two partitions.

To test whether Pol II TSS peak data satisfy the requirement of being informative for most genes, we classified genes into three categories based on their isoforms’ Pol II TSS peak status. Since isoforms that have TSSs close to each other often share the same Pol II TSS peak and thereby the same Pol II prior, we clustered them into ‘isoform TSS groups’ if they were from the same gene and had their TSS within 500 nt of each other. Based on the Pol II peak status of their isoform TSS groups, we classified genes into three categories: (1) “all,” if all of a gene's isoform TSS groups had peaks; (2) “mixed,” if only some groups had peaks; and (3) “none,” if no group had a peak (Fig. 2C). In the K562 data set, 54.2% of expressed protein-coding genes fell into the “mixed” category and did not share RNA-seq reads with any other genes (Fig. 2D). For these genes, Pol II information is informative in allocating RNA-seq multimapping reads between isoform TSS groups with peaks and those without. At the gene level, Pol II peaks facilitate multimapping read allocation for 5.3% of expressed protein-coding genes (3.4% “mixed,” 1.2% “all,” and 0.7% “none”) (Fig. 2D). In total, at least 59% of all expressed protein-coding genes benefit from Pol II peak information in the estimation of their abundances.

### pRSEM improves quantification accuracy at the isoform level

**Figure 3.**
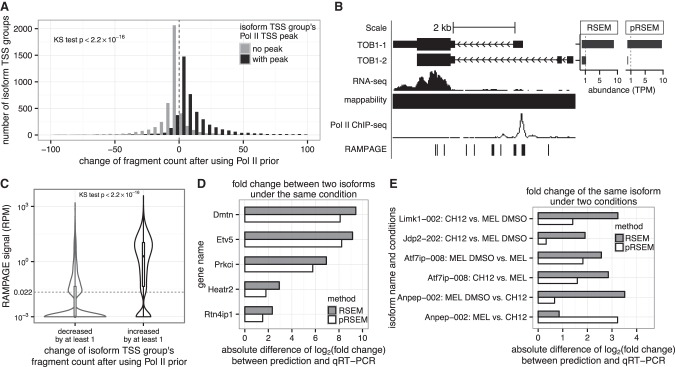
pRSEM more accurately allocates multimapping reads between isoforms. (*A*) Distributions of the change of fragment count between pRSEM and RSEM for isoform TSS groups with (black) and without (gray) Pol II TSS peaks. The two distributions are significantly different (*P* < 2.2 × 10^−16^, Kolmogorov-Smirnov test). (*B*) An example of multimapping read allocation. Shown are *TOB1*’s two protein-coding isoforms, mappability, RNA-seq signal, Pol II ChIP-seq signal, RAMPAGE signal, and estimated abundances from RSEM and pRSEM (*top right*). (*C*) Distributions of RAMPAGE signals for isoform TSS groups that have their fragment counts decreased by at least one (gray) or increased by at least one (black) after using a Pol II–informed prior. The dashed line denotes the signal corresponding to one RAMPAGE read. The two distributions are significantly different (*P* < 2.2 × 10^−16^, Kolmogorov-Smirnov test). A small fractional number (10^−3^) was added to the RAMPAGE signal for each isoform TSS group to allow display of zero signals on a log scale. Data shown in *A* through *C* are based on the K562 RNA-seq replicate one and RAMPAGE replicate one data sets. Isoform TSS groups in *A* and *C* are from genes that had “mixed” peak status and did not overlap or share reads with any other gene. Isoform TSS groups that were estimated to have abundance of less than one TPM according to both RSEM and pRSEM or that had fragment count changes between the two methods of less than one or larger than 100 were excluded. (*D*) Comparison of RSEM (gray) and pRSEM (white) estimated fold changes between a gene's two isoforms in the MEL cell line with fold changes measured by qRT-PCR. (*E*) Comparison of RSEM (gray) and pRSEM (white) estimated isoform fold changes between two conditions with fold changes measured by qRT-PCR.

We first evaluated pRSEM at the isoform level. We compared isoform TSS group fragment counts estimated by pRSEM and RSEM from the K562 data (Supplemental Table S1, replicate one). Fragment counts for a large number of isoform TSS groups with Pol II peaks increased after using pRSEM, whereas the majority of isoform TSS groups without Pol II peaks had decreased fragment counts (Fig. 3A). Distributions of the change of fragment counts for these two types of isoform TSS groups were significantly different (Kolmogorov-Smirnov test, *P*-value <2.2 × 10^−16^). Similar differences were observed in other human and mouse cell lines (Supplemental Fig. S7), indicating that pRSEM allocates reads from isoforms without a Pol II TSS peak to those with peaks relative to RSEM's read allocation.

The abundance estimates in K562 for the isoforms of the gene transducer of ERBB2, 1 (*TOB1*) provide a good example of pRSEM's improved multiread allocation at the isoform level (Fig. 3B). *TOB1* has two alternatively spliced protein-coding isoforms, the TSSs for which are separated by >1.5 kilobases. In K562, only *TOB1-1* has a Pol II peak at its TSS. RSEM estimates that both isoforms have abundances greater than one transcript per million (TPM). Since one TPM is a common cutoff for determining if an isoform is expressed or not, RSEM's result suggests that both isoforms are expressed. In contrast, pRSEM estimates that only *TOB1-1* is expressed, with *TOB1-2* having abundance close to zero TPM. This difference in the estimated expression status of *TOB1-2* has potentially important biological implications because *TOB1* is a tumor suppressor (Kundu et al. 2012), and in gastric cancer cells, miR-25 represses *TOB1* by binding to a 3′-UTR region that is present in *TOB1-1* but absent in *TOB1-2* (Li et al. 2015). Since the *TOB1* gene does not share any reads with other genes, this example demonstrates again that pRSEM allocates reads from isoforms without a Pol II TSS peak to those with a peak relative to RSEM's read allocation. Moreover, pRSEM's estimates agree with RAMPAGE, which is an independent data set characterizing transcriptional activity at the 5′ ends of isoforms. *TOB1-1* has strong RAMPAGE signals at its TSS, whereas *TOB1-2* has almost none, also indicating that only *TOB1-1* is expressed and *TOB1-2* is not. This consistency reveals pRSEM's strength in reducing the number of “false-positive” isoforms, i.e., isoforms called as expressed that truly are not.

We further validated pRSEM by examining RAMPAGE signals (Batut et al. 2013) for all isoforms. We divided isoform TSS groups into those that had fragment counts increased by at least one after we used pRSEM and those had fragment counts decreased by at least one. In K562, the distributions of RAMPAGE signals for these two sets were significantly different (Kolmogorov-Smirnov test, *P*-value <2.2 × 10^−16^) (Fig. 3C). The “increased” groups populated above one read-per-million (RPM). In contrast, the majority of the isoforms in the “decreased” group did not have any RAMPAGE signal, and a small fraction of them had a signal distributed around 0.022 RPM, which corresponded to a single RAMPAGE read. Similar consistency between pRSEM and RAMPAGE was also observed in human cell line GM12878 (Supplemental Fig. S8). These agreements show that pRSEM allocates reads in the right direction.

We next employed qRT-PCR experiments to assess the accuracy of isoform fold change estimates from pRSEM and two comparable methods, RSEM and eXpress. Two sets of validation experiments were performed, one measuring abundance fold difference of two different isoforms of the same gene within a single condition and the other measuring abundance fold change of the same isoform across two different conditions. For the validation within a single condition, pairs of isoforms of the same gene were selected with the criteria that one isoform had a Pol II TSS peak and had a fragment count increase of at least one after we applied the Pol II prior, and the other did not have a Pol II TSS peak and had its fragment count decrease by at least one after application of the prior (Supplemental Data S1; for detailed selection criteria, see section III.C in the Supplemental Material). We compared fold differences measured by qRT-PCR (Supplemental Data S2; Supplemental Table S4) with those estimated by the three quantification methods from RNA-seq data. The log_2_ fold differences estimated by pRSEM were closer to those measured by qRT-PCR than estimates from either RSEM or eXpress for all five pairs of isoforms (Fig. 3D; Supplemental Fig. S10A,C). Both pRSEM and RSEM consistently underestimated the fold differences, whereas eXpress either greatly overestimated the fold differences or estimated little difference between the abundances of each pair of isoforms (Supplemental Data S2). When additionally comparing to estimates from variants of RSEM and eXpress (for the definitions of variants, see section I.A in the Supplemental Material), pRSEM estimates had the smallest differences to qRT-PCR measurements for three out of five pairs of isoforms (Supplemental Fig. S10A,C). Scatterplots of the estimated versus qRT-PCR measured abundances of the individual isoforms revealed that although the pRSEM and RSEM estimates correlated similarly with qRT-PCR, pRSEM had smaller estimates for the low-abundance isoforms, which resulted in more accurate fold differences (Supplemental Fig. S9). We conjecture that the relatively poor performance in fold difference estimation by all methods was due to the selection criteria for this experiment, which required that the low-abundance, Pol II peak-lacking isoform had a nonnegligible estimated abundance by pRSEM, such that qRT-PCR would likely succeed. These criteria may have selected for cases in which pRSEM overestimated the abundance of a Pol II peak-lacking isoform, and thus these criteria were modified for the second type of experiments.

In the second set of validation experiments, we evaluated the accuracy of single isoform fold change estimates across two conditions. We selected isoforms that had a Pol II TSS peak in one condition, but not in another, and for which the abundance estimates from RSEM and pRSEM differed by a factor of two in one condition (Supplemental Data S3; for detailed selection criteria, see section III.C in the Supplemental Material). These criteria resulted in isoforms with fold change estimates that were markedly different between the methods. As before, we compared the estimated log_2_ fold changes with qRT-PCR measurements (Supplemental Data S4; Supplemental Table S4). In five out of six cases, pRSEM's estimates were closer to the qRT-PCR measurements than RSEM's (Fig. 3E). Moreover, all fold changes from pRSEM were better than those from eXpress and variants of RSEM and eXpress (Supplemental Fig. S10B,D; Supplemental Data S4). The closer agreement between pRSEM and qRT-PCR in these experiments illustrates pRSEM's advantages over other quantification methods. Because our qRT-PCR experiments only considered isoforms with varying Pol II peak status across conditions, we additionally performed data-driven simulations to examine the accuracy of quantifications of isoforms for which Pol II information is not explicitly informative. Interestingly, we found that pRSEM also outperforms RSEM for these isoforms (Supplemental Fig. S15).

Given pRSEM's higher accuracy over RSEM with respect to isoform abundance estimation, we went on to explore broader biological implications of the isoform quantifications obtained with pRSEM. We first performed a genome-wide survey of active TSSs in human and mouse cell lines. For all of the five cell lines examined, more than 700 TSSs were revealed to be active by RSEM, but not by pRSEM (Supplemental Table S5), suggesting that RSEM overestimates the numbers of active TSSs. We next examined expressed isoforms in primary cells at various stages of mouse hematopoietic differentiation (Lara-Astiaso et al. 2014). pRSEM identified a much smaller number of expressed isoforms (and genes) than RSEM for all 16 cell types (Supplemental Table S6). Lastly, differential expression analysis using EBSeq (Leng et al. 2013) on three mouse cell lines with RSEM and pRSEM estimates revealed more than 2000 isoforms that differed in their differential expression call between the two methods (Supplemental Table S7). All three surveys suggest that pRSEM presents a different genome-wide isoform profile than RSEM, and such a difference is highly likely to lead to novel functional characterizations.

### pRSEM identifies RSEM-misclassified unexpressed genes

**Figure 4.**
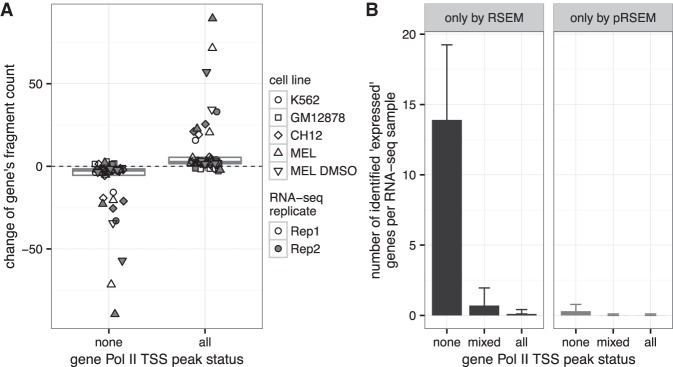
pRSEM more accurately allocates multimapping reads between genes. (*A*) Changes of estimated fragment counts between pRSEM and RSEM for pairs of genes overlapping with each other and sharing reads exclusively. Genes that had a fragment count change of less than one were excluded. (*B*) The numbers of genes for which RSEM (black) and pRSEM (gray) disagreed on expression status. Genes considered were those sharing RNA-seq reads with others but not overlapping with any other genes. The numbers were calculated from 10 RNA-seq replicates from five human and mouse cell lines. Error bars represent one standard deviation.

At the gene level, we first investigated how pRSEM allocates reads between overlapping genes (Fig. 1A, panel II). We built a data set that contained pairs of overlapping genes that shared reads exclusively. For each pair of genes, we required that one gene had a Pol II peak status of “all” and the other had a peak status of “none” (Fig. 2C). We compared the change of the fragment counts for these pairs of genes between RSEM and pRSEM and selected those that changed by more than one. We observed, for both human and mouse cell lines, that most of the “none” genes had fragment counts decreased after we used a Pol II prior, whereas most of the “all” genes had fragment counts increased (Fig. 4A). Since fragments could only be transferred between the two genes within each pair, the two different distributions show that pRSEM correctly allocates fragments from genes without a Pol II peak to those with Pol II peaks.

Next, we examined how pRSEM performs on allocating reads between nonoverlapping genes (Fig. 1A, panel III). Since genes often share reads with multiple nonoverlapping genes with different Pol II peak status, building a data set of pairs of genes that shared reads exclusively (as we did for overlapping genes) was not feasible. Instead, we compared expression state calls (i.e., “expressed” or “unexpressed”) of nonoverlapping genes between RSEM and pRSEM. The two methods agreed for a majority of the genes. On average, 98% of human and mouse genes’ expression states were called the same if one TPM was used as the “expressed” cutoff (Supplemental Table S8). For genes on which RSEM and pRSEM disagreed in terms of expression state, we inspected the methods’ agreement with the Pol II peak data, with the assumption that genes with Pol II peak status of “mixed” or “all” are expressed and those of type “none” are unexpressed. Along this line, we define expressed “none” genes as false positives and unexpressed “mixed” or “all” genes as false negatives. Across five human and mouse cell lines, on average, pRSEM eliminates 14 of RSEM's false positives, while introducing fewer than one false positive or false negative of its own (Fig. 4B). This contrast between the number of misclassifications removed and the number of misclassifications added held true when we decrease the “expressed” cutoff to 0.5 TPM or increased it to 2 TPM (Supplemental Fig. S12), illustrating pRSEM's strength in identifying false-positive genes. Moreover, pRSEM maintains this advantage when compared with eXpress variants (Supplemental Fig. S13).

### pRSEM has a lower false-positive rate than alternative methods in data-driven simulations

**Figure 5.**
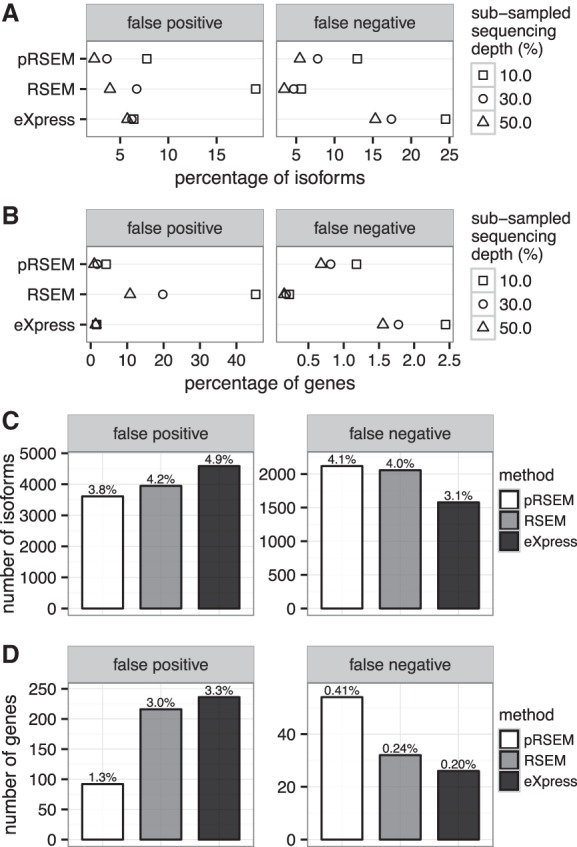
Data-driven simulations reveal that pRSEM has a lower false-positive rate (FPR) than RSEM and eXpress. (*A*,*B*) Shape plots (squares, circles, triangles) represent FPR and false negative rate (FNR) for isoforms (*A*) and genes (*B*) in subsampling experiments. (*C*,*D*) Bar plots show the number of isoforms (*C*) and genes (*D*) classified as false positives and false negatives from simulations at full sequencing depth. FPR and FNR are shown *above* each bar. A false positive is an isoform or gene with a true abundance <1 TPM and an estimated abundance ≥1 TPM. A false negative is an isoform or gene with a true abundance ≥1 TPM and an estimated abundance <1 TPM.

To comprehensively evaluate pRSEM, we carried out two types of data-driven simulations. The first type consisted of subsampling experiments in which we performed quantification on random samples of 10%, 30%, and 50% of the reads in the K562 replicate one data set. Read depth for these samples ranged from 6.8 million to 33.8 million (Supplemental Table S9). The abundance estimates from the subsamples were then compared with those obtained from the entire data set using RSEM's maximum likelihood estimates, which we took to be the “truth.” We evaluated pRSEM, RSEM, and eXpress in terms of false-positive rate (FPR) and false-negative rate (FNR). We define FPR as the percentage of unexpressed isoforms or genes that are predicted to be expressed and FNR as the fraction of expressed isoforms or genes that are predicted to be unexpressed. At the isoform level, pRSEM always had lower FPR but higher FNR than RSEM (Fig. 5A). RSEM's lower FNR could be attributed to the larger pseudofragment count assigned uniformly to every isoform (corresponding to an uninformative Dirichlet prior), which slightly boosts their expression levels. When we used a much smaller uniform prior parameter (0.049–0.053 under different subsampled sequencing depths) learned from a single partition, the estimates had FNR comparable to pRSEM (Supplemental Fig. S14A). Compared with eXpress, pRSEM had comparable or lower FPR and markedly lower FNR (Fig. 5A). At the gene level, the relative performances of the methods were similar to those at the isoform level (Fig. 5B; Supplemental Fig. S14B). Additional batch EM rounds of eXpress did not change its qualitative differences from pRSEM (Supplemental Fig. S14A,B). Together, these subsampling experiments indicate that compared with RSEM and eXpress, pRSEM produces estimates with a favorable balance of FPR and FNR.

We performed a second type of simulation in which we generated a synthetic read data set with the same sequencing depth as the full K562 data set. We drew each isoform's fragment-generating probability (θ in RSEM's probabilistic model) from a Pol II–derived Dirichlet distribution (Fig. 2B). Then, based on all isoforms’ fragment-generating probabilities and effective lengths, we calculated isoform abundances and applied RSEM's simulator to generate RNA-seq reads. At the isoform level, pRSEM had a 0.4% lower FPR and 0.1% higher FNR than RSEM (Fig. 5C). Again, RSEM's better FNR could be attributed to its use of an uninformative uniform prior (Supplemental Fig. S14C). Compared with eXpress, pRSEM had a 1.1% lower FPR and a 1.0% higher FNR (Fig. 5C). In absolute terms, pRSEM had 974 fewer false-positive isoforms and 540 more false-negative isoforms, indicating that pRSEM's lower FPR comes at the expense of a smaller number of false-negative isoforms. At the gene level, pRSEM's advantage with respect to FPR was even more evident: pRSEM's FPR was ∼2% less than those of RSEM and eXpress (Fig. 5D). Further, a naïve approach using RSEM fragment counts cannot eliminate false-positive isoforms or genes called solely by RSEM (Supplemental Fig. S16). All three methods, as well as their variants had low FNR at the gene level (all <0.5%), with eXpress having the lowest FNR (Fig. 5D; Supplemental Fig. S14D). Again, additional batch EM rounds of eXpress did not change its qualitative differences from pRSEM (Supplemental Fig. S14C,D). In summary, simulations of synthetic data for which we know the true abundances of isoforms illustrate pRSEM's strength at reducing the number of false positives compared with RSEM and eXpress.

### Informative priors for pRSEM can be learned from a broad range of data types

To characterize the general applicability of pRSEM, we first asked whether Pol II ChIP-seq data from a different cell line could be informative for RNA-seq quantification.

**Figure 6.**
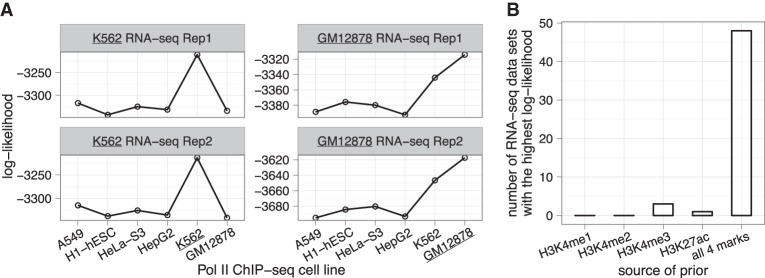
Informative priors for pRSEM can be derived from a broad range of data types. (*A*) Comparison of training set log-likelihoods based on isoforms partitioned by Pol II ChIP-seq peaks from six human cell lines. Log-likelihoods were computed by fitting pRSEM's Dirichlet-multinomial model to RNA-seq fragment counts of the partitioned training set isoforms. (*B*) Comparison of five sources of prior information by their effectiveness on 52 RNA-seq data sets of mouse hematopoietic cells. For each RNA-seq data set, all five sources were separately applied to fit the training set and the goodness of fit was assessed via the log-likelihood. The source resulting in the largest log-likelihood was considered to be the most effective. “All 4 marks” denotes a pRSEM partition model utilizing all four types of histone modification signals.

We obtained Pol II peaks from six different human cell lines (Supplemental Table S2) and applied them to the RNA-seq data from the K562 and GM12878 cell lines (Supplemental Table S1). As expected, Pol II peak information from the same cell line as the RNA-seq data always gave the best fit to the training set isoforms’ fragment counts (Fig. 6A), suggesting that complementary data sets from matched conditions are the most effective sources of prior. Surprisingly, Pol II peaks from any of the six cell lines were able to partition the training set isoforms into two groups with significantly different fragment count distributions (Supplemental Fig. S5), indicating that Pol II data from a different cell line is also informative. However, the extent to which a complementary data set from an unmatched condition could benefit RNA-seq quantification was not examined.

Next, we investigated if histone modification ChIP-seq data could also inform RNA-seq multimapping read allocation. Histone modification signals have been shown to correlate with gene expression levels (Karlić et al. 2010; Dong et al. 2012), and a trimodal abundance distribution similar to the one based on Pol II ChIP-seq data (Fig. 2B) was previously observed when genes were partitioned by histone marks (Hebenstreit et al. 2011). To specifically investigate whether histone data could be informative for pRSEM's model, we used data from a study of mouse hematopoietic differentiation for which each sample had associated RNA-seq and histone ChIP-seq data, but no Pol II data (Lara-Astiaso et al. 2014). Each of the 16 cell types had ChIP-seq data collected by the same protocol for four types of histone modifications: H3K4me1, H3K4me2, H3K4me3, and H3K27ac. For each histone modification, we used the presence or absence of a ChIP-seq peak within an isoform's TSS region to partition isoforms in the training set. All four marks in all 16 cell types were found to partition the isoforms into two groups that had significantly different fragment count distributions (Supplemental Fig. S6A), suggesting that histone modification ChIP-seq data can serve as an informative prior for pRSEM. Therefore, when Pol II ChIP-seq data are not available for the cells being assayed by RNA-seq, a variety of other data, such as histone ChIP-seq or Pol II ChIP-seq from other cell lines, may still be used effectively by pRSEM.

We implemented several tools in pRSEM to assist users in selecting the appropriate external sources of data. First, we provided a testing procedure to determine whether an external data set is informative and in the case that multiple data sets are informative, which one is best (section II.F in the Supplemental Material). As an alternative to selecting a single best prior-informing data set, we developed a partition model that employs logistic regression to combine multiple informative signals (section II.B in the Supplemental Material). For the mouse hematopoietic differentiation data sets, we found that using ChIP-seq data from all histone marks together outperformed using any one of the marks individually (Fig. 6B; Supplemental Fig. S6B).

## Discussion

We have developed a new computational method named pRSEM that utilizes ChIP-seq data in the task of quantifying transcripts from RNA-seq data. We have shown that pRSEM can allocate multimapping reads properly according to Pol II information at both the isoform and gene level. As a result, abundance estimates from pRSEM are more accurate than those from RSEM and eXpress, as validated by qRT-PCR.

Compared with existing methods, the main advantage of pRSEM is its improved quantification of low-abundance or unexpressed genes and isoforms. This advantage was illustrated by pRSEM's superior low FPR in data-driven simulations. Correctly identifying expressed isoforms and genes is an important aspect of RNA-seq quantification, because such information is the basis for understanding upstream and downstream regulatory effects. Moreover, a large fraction of isoforms and genes are generally expressed at low to moderate levels. According to RSEM, pRSEM, and eXpress's estimates, 38%–55% of isoforms and 23%–38% of genes have abundances in the range 0.1–10 TPM (Supplemental Fig. S11). Thus, better estimation of the abundances of these isoforms and genes can have a large effect on overall quantification accuracy. There is less information in an RNA-seq data set with which to estimate the abundances of isoforms and genes in this range. We have shown that pRSEM effectively leverages external information, such as Pol II and histone ChIP-seq data, to address this critical barrier.

The Dirichlet-multinomial model used by pRSEM provides a flexible framework for the incorporation of prior information from a variety of sources. Users of pRSEM may derive priors from transcription factor or histone modification ChIP-seq data in addition to many Pol II ChIP-seq data sets. All of pRSEM's partition models are designed in a way that does not depend on any specific ChIP-seq target. Moreover, other types of transcriptional data, such as CAGE, GRO-seq, RAMPAGE, and 5′-RACE (Core et al. 2008; Hoskins et al. 2011; Batut et al. 2013; The FANTOM Consortium and the RIKEN PMI and CLST (DGT) 2014), can serve in the derivation of a pRSEM prior as well. This adaptive feature of pRSEM makes it applicable to a broad range of RNA-seq data.

Interestingly, the Poll II–derived prior parameters, which can be interpreted as pseudofragment counts, are relatively small (Supplemental Fig. S4). For example, the prior parameters estimated from the K562 replicate one data set were 0.60 and 0.04 for the “with peak” and “no peak” partitions, respectively. Such small pseudofragment counts play a minor role in multiread allocation when genes or isoforms have a large number of uniquely mapped reads. This likely explains why we did not observe dramatic changes in estimated abundances between RSEM and pRSEM for highly expressed genes or isoforms (Supplemental Fig. S11). Increasing sequencing depth, which increases the number of uniquely mapped reads, remains an effective way to resolve the multimapping issue as indicated by our subsampling experiments (Fig. 5A,B). However, due to alternative processing, >45% of human expressed genes have indistinguishable isoforms (Fig. 1B), and their abundances cannot be accurately estimated solely based on uniquely mapped reads. Therefore, Pol II and histone modification ChIP-seq data represent invaluable sources for guiding multiread allocation of these isoforms.

Although we have found that many condition-matched (and even unmatched) external data sets are informative for deriving a pRSEM prior, further work will be needed to determine the degree to which an external data set must be statistically informative in order for it to be practically useful for RNA-seq quantification. Through our experiments on both human and mouse data, we have provided evidence that matched Pol II ChIP-seq data can allow for marked improvements in RNA-seq quantifications. However, there may be situations in which Pol II ChIP-seq data may not be practically useful. For example, if the ChIP-seq data are of poorer quality than those analyzed in this work, isoforms will be more likely to be misclassified in terms of their Pol II peak status. In addition, the strength of a Pol II–derived prior depends on the extent to which transcription is the primary determinant of expression levels. When post-transcriptional effects, such as differential isoform degradation, play a comparable role in determining expression levels, such a prior may not be as useful. To remedy this latter issue, complementary data sets providing information regarding post-transcriptional effects could be used in combination with Pol II ChIP-seq data to derive a prior for pRSEM.

To our knowledge, pRSEM is the first RNA-seq quantification method to use a complementary data set relevant to the sample of interest and, as such, builds a foundation for integrative quantification methodology. Through its use of an additional data type, pRSEM enables more accurate quantification than can possibly be achieved by RNA-seq data alone. We expect that integration of data from other high-throughput technologies in addition to Pol II and histone ChIP-seq data will continue to advance the limits of transcript quantification.

## Methods

The framework of pRSEM is built on RSEM, which employs a generative model and an EM algorithm to estimate gene and isoform expression levels. RSEM also includes a Bayesian formulation of its model, in which transcript expression levels are viewed as latent variables from a Dirichlet distribution. In this Bayesian mode, RSEM uniformly sets the prior parameters for the Dirichlet distribution to one (an uninformative prior) so that the maximum a posteriori estimates are equal to RSEM's maximum likelihood estimates. The framework of pRSEM takes advantage of this design and instead learns informative parameters for the Dirichlet prior using a partitioned training set. In this way, pRSEM can bring in external information to supervise the allocation of multimapping reads and estimate transcript abundances. For any given external data set, pRSEM first builds a training set of isoforms and partitions them based on the external data. A single shared prior parameter is learned for each partition through maximization of the likelihood of a Dirichlet-multinomial model, with the read counts of the training set isoforms as data. These prior parameters are then used during the quantification of all isoforms, which are partitioned in the same manner using the external data. A detailed description of pRSEM is in the Supplemental Material section II.

RNA-seq and ChIP-seq data sets were obtained from two sources. One source was ENCODE (The ENCODE Project Consortium 2012; Mouse ENCODE Consortium et al. 2012), from which data were obtained for five human and mouse cell lines: K562, GM12878, CH12, MEL, and MEL treated with 2% DMSO for 5 d (referred to as “MEL DMSO”). All cell lines had associated RNA-seq data and ChIP-seq data of Pol II and control (Supplemental Table S1 and S2). In addition, we obtained Pol II ChIP-seq peak files for another four human cell lines (Supplemental Table S2) for our experiments regarding the informativeness of unmatched Pol II data. The other source of RNA-seq and ChIP-seq data used in our experiments was a mouse hematopoietic differentiation study (Lara-Astiaso et al. 2014) (Gene Expression Omnibus accession numbers GSE60101 and GSE59636). All cell types assayed in this study had associated RNA-seq data and ChIP-seq data for four histone modifications (H3K4me1, H3K4me2, H3K4me3, and H3K27ac).

Transcript annotations were taken from GENCODE human version 19 and mouse version 4 (Harrow et al. 2012). UCSC genome assemblies hg19 and mm10 were used for human and mouse, respectively. RNA-seq reads were aligned by STAR v2.4.0h (Dobin et al. 2012) and quantified by pRSEM, RSEM v1.2.15 (Li and Dewey 2011), or eXpress v1.5.1 (Roberts and Pachter 2013). ChIP-seq reads for Pol II and its control were aligned with Bowtie v1.0.1 (Langmead et al. 2009), and peaks were called by ENCODE's SPP and IDR pipeline (Landt et al. 2012) with an IDR threshold of 0.05. Due to the lack of ChIP-seq controls for the mouse hematopoietic cell samples, peaks for these samples were called by HOMER, as described previously (Lara-Astiaso et al. 2014). The command line options used for each software are described in section I.A and section I.C of the Supplemental Material.

Multiple variants of pRSEM, RSEM, and eXpress were compared in this work. “pRSEM” refers to a pRSEM run under its default two-partition scheme. “pRSEM no partition” represents a pRSEM run where a single prior parameter is learned for all isoforms in the training set without a partition. “RSEM” refers to the posterior mean estimates obtained from Gibbs sampling with the Bayesian version of RSEM's probabilistic model with an initial pseudocount of one for every isoform. “RSEM” is the most comparable variant to pRSEM. “RSEM ML” refers to the maximum likelihood estimates obtained from RSEM's expectation-maximization algorithm. “eXpress” denotes an eXpress run under default settings. “eXpress O1B10” and “eXpress O1B100” denote an eXpress run with one round of online EM, followed by 10 or 100 rounds of batch EM, respectively.

An isoform group's RAMPAGE signal was defined as the read-depth-normalized number of RAMPAGE reads that have their 5′ ends map within the 100-nt flanking regions of the TSS of any isoform in this group. Two types of qRT-PCR validations were carried out. The first one measured the fold change of expression level between two isoforms from the same gene under the same condition. The other compared the fold change of expression level for the same isoform under two different conditions. Details on isoform selection and qRT-PCR measurement are described in section III.C of the Supplemental Material.

Reads for subsampling experiments were randomly selected from 10%, 30%, and 50% of K562 RNA-seq replicate one. RSEM ML estimates on K562 RNA-seq replicate one were used as the ground truth. The number of reads and noise parameter for simulation at full-sequencing depth were based on K562 RNA-seq replicate one as well. All isoforms were partitioned by their K562 Pol II ChIP-seq peak status, and the isoforms’ fragment-generating probabilities were drawn from the distribution learned from the training set isoforms. Details on the two types of experiments are provided in section IV of the Supplemental Material.

### Software availability

The source code of pRSEM is available in the Supplement. The latest version of pRSEM and a demo can be found at https://deweylab.github.io/pRSEM/.
